# Urban density and COVID-19: understanding the US experience

**DOI:** 10.1007/s00168-022-01193-z

**Published:** 2022-11-28

**Authors:** Felipe Carozzi, Sandro Provenzano, Sefi Roth

**Affiliations:** Department of Geography and Environment, London School of Economics, London, UK

**Keywords:** I12, R12

## Abstract

This paper revisits the debate around the link between population density and the severity of COVID-19 spread in the USA. We do so by conducting an empirical analysis based on graphical evidence, regression analysis and instrumental variable strategies borrowed from the agglomeration literature. Studying the period between the start of the epidemic and the beginning of the vaccination campaign at the end of 2020, we find that the cross-sectional relationship between density and COVID-19 deaths changed as the year evolved. Initially, denser counties experienced more COVID-19 deaths. Yet, by December, the relationship between COVID deaths and urban density was completely flat. This is consistent with evidence indicating density affected the timing of the outbreak—with denser locations more likely to have an early outbreak—yet had no influence on time-adjusted COVID-19 cases and deaths. Using data from Google, Facebook, the US Census and other sources, we investigate potential mechanisms behind these findings.

## Introduction

Historically, cities have been associated with the propagation of infectious diseases.^1^ It is therefore not surprising that the impact of density—the defining feature of cities—on the spread of COVID-19 was a frequent talking point from the very outset of the COVID-19 pandemic. As early as 22nd of March 2020, in the context of a critical outbreak in New York City, state governor Andrew Cuomo tweeted “There is a density level in NYC that is destructive. It has to stop and it has to stop now. NYC must develop an immediate plan to reduce density.”^2^

The notion that dense cities would be hotbeds of virus transmission prompted a flurry of academic research on the topic. Initial studies—especially those looking at the United States’ experience—suggested urban density fostered a faster spread of the disease.^3^ Similar evidence was reported for other countries including India (Bhadra et al. 2021), Brazil (Pequeno et al. 2020) and Germany (Ehlert 2021). However, subsequent research exploring a longer time series yielded mixed findings (see for example McFarlane 2021; Kim et al. 2021; Florida et al. 2021). This prompted a more nuanced approach to the question, and subsequent work on the roles of crowding, experienced density and other more direct measures of social interactions.^4^

Now that massive vaccination campaigns have gradually reduced the threat of COVID-19 worldwide, can we draw any definitive conclusions about the mediating role of density in shaping the health impact of COVID-19 in cities? We turn to this question by looking at the evolution of the epidemic in the contiguous USA, in the period between the first registered cases in January 2020 and the beginning of the vaccination campaign in mid-December. By looking at the whole of 2020, we seek to understand how the results of initial studies indicating density was an important determinant of the impact of COVID-19 progressively led to more ambiguous findings as the pandemic evolved. Our empirical analysis combines descriptive evidence with an instrumental variable strategy borrowed from the agglomeration literature in economics. In doing so, our methodological approach avoids some of the pitfalls of conventional regression estimates and is close to methods that are familiar to both economists and economic geographers.

We find convincing evidence that density affected the timing of the outbreak in each county, with denser locations more likely to have an early outbreak. We show this leads to an initially positive and significant relationship between the impact of COVID-19 and population density at the county level, consistent with the results of early studies on the spread of the virus in the USA. However, after adjusting for the timing of the onset of the disease in each county, we find no evidence that population density is positively associated with the impact of COVID-19. Interestingly, we find a negative relationship between density and the spread of COVID-19 within a county at the very beginning of an outbreak, but this relationship fades completely within 2 months. We also show that, by the end of 2020, density could no longer explain the cross-sectional pattern of accumulated cases or deaths. Dense locations were hit first, but, as the pandemic evolved, they were not hit harder. These results help us frame other studies on this topic and understand how the findings in that literature changed as the pandemic developed.

The fact that—by the end of 2020—density had no effect on the local impact of COVID-19 appears counter-intuitive. The virus spreads via human contact and denser areas provide more opportunities for human interaction. Yet, this is not the only way in which density can affect the spread of disease. Several mediating factors can make the direction of this relationship theoretically ambiguous. We analyze social/behavioral factors that could explain our findings, bearing in mind that the spread of disease is a social as well as a biological phenomenon (Papageorge et al. 2020). To do so we use data from Google, Facebook, the US Census and The County Health Rankings and Roadmaps program. First, we show that density is positively associated with the reduction in work- and leisure-related activities throughout the pandemic, suggesting that compliance with social distancing measures was higher in denser locations. Second, we use our empirical strategy to illustrate the well-known fact that density is negatively associated with the share of Republican voters, which have been shown to be less engaged in social distancing and other efforts to reduce transmission (Allcott et al. 2020). Third, we show population density is positively associated with access to healthcare and income and negatively associated with inhabitants’ age. Collectively, these results yield suggestive evidence of mechanisms generating offsetting negative effects of density on the spread and severity of the COVID-19 outbreak, and help us rationalize the estimates of the overall effects reported in our main analysis.

Estimating how population density shaped the spread and severity of the COVID-19 outbreak, as well as its effects on local behavioral responses and demographics is challenging for several reasons. First, population densities are not randomly assigned and they might be correlated with unobserved confounding factors. For example, population densities can be affected by locational productive advantages, whether natural or man-made (e.g., soil quality or transportation infrastructure), that may also simultaneously affect local economic conditions. Insofar as the COVID-19 outbreak is affected by economic factors, unobservable locational advantages can confound the effect of density on the spread and severity of the disease. Second, differences in the timing of the onset of the disease can generate cross-sectional differences in the severity of the outbreak at one point in time in the absence of true differences in the local reproduction rate. Finally, data on COVID-19 cases might be reported with error due to variation in local testing strategy and capacity.

We overcome the empirical challenges mentioned above in several ways. We use two Instrumental Variable (IV) strategies borrowed from the agglomeration literature in economics to induce plausibly exogenous variation in population density without affecting COVID-19 cases and deaths directly. More specifically, in our *geological* IV approach, we use the presence of aquifers, earthquake risk, and soil drainage capacity to as instruments for density (as in Duranton and Turner 2018). In our *historical* IV strategy, we use the traditional long-lag instrument, which measures urban population density in the 1880 US Census (as in Ciccone and Hall 1996 and a large subsequent literature). We use these tools to study both how density affected the timing of the outbreak in each county and the time-adjusted number of deaths after that outbreak. We focus on the daily number of confirmed COVID-19 *deaths* rather than cases as our main outcome of interest since this is considered to be a more accurate indicator of local COVID-19 prevalence (Subbaraman 2020), and discuss COVID-reported cases as a robustness check. Finally, we cross-validate our COVID-19 figures with official data from the CDC to ensure reported deaths are consistent with other measures of COVID-19 mortality.

As discussed above, a number of papers have examined the link between density and COVID-19 incidence in the USA.^5^ Alongside these studies, a vast number of papers in economics and economic geography have focused on other social determinants of differences in the spread of COVID-19 such as mobility Glaeser et al. (2020), Almagro et al. (2020), racial composition Benitez et al. (2020), Hamman (2021), social capital and institutions Ding et al. (2020), Rodríguez-Pose and Burlina (2021) as well as on the predicted long-run impact of the pandemic on cities (Florida et al. 2021), Nathan and Overman (2020). We contribute to this literature by looking specifically at density—arguably one of the first explanatory factors that attracted the attention of the field in early 2020—and its changing role throughout the US epidemic. Given that density is associated with many of the factors that were studied subsequently—mobility, race, urbanization—our findings also help interpret the results reported in the broader literature.

## Data

Our dataset combines information on COVID-19 cases and deaths, population density, demographics, social connectedness, behavioral changes, voting behavior, healthcare provision, income and geological features at the US county level. We will use COVID data extending over the period between the 22nd of January, when the first US case was confirmed in King County, up until the 15th of December 2020, the day after the COVID vaccination campaign began in the USA. We restrict our sample to urban counties^6^ in the contiguous USA which leaves us with 1759 counties comprising $\sim$ 93% of the total US population. When analyzing the pace of the outbreak, we further restrict the sample further to those counties that had at least one confirmed COVID-19- related death 60 days before the end of our sample period. This *Outbreak Sub-sample* consists of 1441 counties representing $\sim$ 89 % of the total US population (see Fig. 4). In the following, we describe the dataset and provide further information about the sources and URLs for download in “Appendix B” and descriptive statistics in Table 1.

**Table 1:** Descriptive statistics

	Mean	Standard deviation
*(A) Full sample*		
Population density	147	696
Weighted population density	522	1117
Population	173,406	432,333
COVID deaths 60 days after 10th case	49	272
COVID cases 60 days after 10th case	841	3376
Share of population above 60 years	0.24	0.05
$\Delta$ workplace-related activity	−40.61	7.82
$\Delta$ retail-related activity	−35.62	11.98
Number of counties	1759	
Share of US population: 93%		
*(B) COVID outbreak sub-sample*		
Population density	173	766
Weighted population density	585	1220
Population	203,190	472,196
COVID deaths 60 days after 10th case	59	299
COVID cases 60 days after 10th case	1008	3704
Share of population above 60 years	0.24	0.05
$\Delta$ workplace-related activity	−41.17	7.92
$\Delta$ retail-related activity	−35.94	11.39
Number of counties	1441	
Share of US population: 89%		

Descriptive statistics presenting the mean and standard deviation for a set of key variables of interest. Panel A corresponds to the whole sample of urban counties (i.e. counties belonging to a CBSA). Panel B corresponds to the Outbreak sub-sample consisting of counties that had at least one confirmed COVID-19 death 60 days before the end of our sample period on the 15th of December 2020

### COVID-19 cases and deaths

We obtain a panel of daily confirmed COVID-19 fatalities and cases for US counties from usafacts.org.^7^ The most intuitive indicator to monitor the COVID-19 outbreak is the daily number of confirmed cases. However, this figure is likely to be distorted by varying local testing strategy and capacity. Furthermore, the ability of the virus to spread across asymptomatic people makes the task of recording the number of infections in the community extremely difficult (Subbaraman 2020). Therefore, we mainly use the daily number of confirmed COVID-19 deaths as this is a more accurate indicator of the local COVID-19 prevalence.^8^ In order to ensure that our COVID-19 data are reliable, we cross-validate our COVID-19 figures with official data from the Centers for Disease Control and Prevention (CDC). In the left panel of Fig. 5, we compare our total COVID-19 fatality counts by county to the latest figures on officially confirmed deaths due to COVID-19. In the right panel, we compare total fatalities to CDC excess death estimates. Both graphs exhibit strong linear relationships and support the validity of our COVID-19 data.^9^ The evolution of daily COVID-19 fatality numbers used in this paper is illustrated in “Appendix Fig. 6.” In our analysis below, when we refer to deaths taking place in the *first-wave*, we refer to those taking place up to the 5th of July, which is the minimum in the moving average of deaths after April 2020.

### Population density

Based on the US census for 2010, we compute two measures of population density. The first is simply the total population of a county over its total area. This will constitute the independent variable of interest throughout most of our analysis. The second variable takes the population density for all census-blocks within a county and computes the associated population-weighted mean. Population-weighted density is meant to measure average “experienced” density and was popularized in economics by Glaeser and Kahn (2004) and Rappaport (2008). It can be computed using spatially disaggregated data on the distribution of population and weighting each small unit of population density by its relative population in the county.

#### Instrumental variables

For our geological instrumental variable estimates, we use three different instruments. More specifically, we use variables measuring earthquake risks and presence of aquifers from the US Geological Survey (USGS) (also used in Duranton and Turner 2018), and data on soil drainage quality from NRCS State Soil Geographic Data Base. We match our grid cells to the geological data using grid cell centroids to spatially impute data on aquifers, earthquake risks and soil drainage quality. For our historical instrument, we use population density obtained from the 1880 US census. We impute these data on the county level using spatial matching based on the assumption of uniform population distribution within 1880 counties.^10^

**Table 2:** Cases and deaths in first COVID-19 wave in 2020: baseline OLS estimates

	Log(cases per 100,000)		Log(Deaths per 100,000)	
Log(population density)	0.218***	0.217***	0.130**	0.126***
	(0.027)	(0.021)	(0.051)	(0.034)
R2	0.20	0.42	0.11	0.36
Obs.	1756	1756	1414	1414

	Log(cases per 100,000)		Log(cases per 100,000)	
Log(weight. density)	0.229***	0.219***	0.150**	0.110***
	(0.027)	(0.020)	(0.059)	(0.036)
State effects	No	Yes	No	Yes
R2	0.21	0.42	0.11	0.35
Obs.	1756	1756	1414	1414

Baseline OLS estimates. Columns (1) and (2) use the log of cases per 100,000, columns (3) and (4) the log of deaths per 100,000 inhabitants as dependent variables, both taken as accumulated by the 5th of July. In the top panel, we report estimates for the effect of log of population density. In the bottom panel, we use the log of population-weighted density. In all models, we include controls for average maximum and minimum temperatures, average yearly precipitation, latitude, longitude, distance between the county centroid and the closest sea front and distance to the closest waterfront. The specifications in columns (2) and (4) add state effects. Standard errors in parenthesis are clustered at the CBSA level. ***${p}<0.01$, **${p}<0.05$, *${p}<0.1$

**Table 3:** Onset of the disease and deaths after 60 days in 2020

	OLS	IV	
*(A) Days to first case*			
Log(population density)	$-$5.957***	$-$4.288***	$-$5.034***
	(0.447)	(0.864)	(0.870)
IV F-stat		25.9	111.0
R2	0.44	0.42	0.43
Obs.	1759	1759	1733
*(B) Days to first fatality*			
Log(population density)	$-$15.935***	$-$6.673**	$-$13.384***
	(1.132)	(3.247)	(2.443)
IV F-stat		24.9	95.3
R2	0.34	0.29	0.33
Obs.	1667	1667	1642
*(C) Log(Deaths per 100,000, 60 days after 10th case)*			
Log(population density)	$-$0.046	$-$0.053	0.043
	(0.040)	(0.125)	(0.077)
IV F-stat		25.6	60.5
R2	0.30	0.30	0.29
Obs.	1441	1441	1418

The main explanatory variable in all models is the natural logarithm of population density. Panels A and B report estimates using the number of days to the first case and first death, respectively, as dependent variables. Panel C reports estimates using the log of the number of deaths per 100,000 residents in a county, 60 days after the 10th reported case as dependent variable. Column (1) corresponds to OLS estimates, and column (2) and (3) presents 2SLS estimates using the Geological and Historical instruments, respectively. In all models, we include controls for average maximum and minimum temperatures, average yearly precipitation, latitude, longitude, distance between the county centroid and the closest sea front and distance to the closest waterfront. The specifications in columns (2) and (3) add state effects. Standard errors in parenthesis are clustered at the CBSA level. ***${p}<0.01$, **${p}<0.05$, *${p}<0.1$

#### Behavioral adjustment/social distancing

To measure how much people in different counties adjusted their behavior as a response to the COVID-19 outbreak, we use the ‘COVID-19 Community Mobility Reports’ by Google (Google CMR). This database aggregates extensive anonymized mobile device GPS user data and estimates the percentage change in activities (such as work, retail or transit) by county and day. The five week period from January 3rd to February 6th before the start of the COVID-19 outbreak in the US serves as the corresponding baseline period.

#### Other variables

We obtain data on county-level demographic characteristic estimates for 2018 from the US census. Social connectedness is measured with Facebook’s Social Connectedness Index (Facebook SCI), which captures the intensity of the link between locations using the number of friend links in this social network (see Bailey et al. 2018 for further details on the SCI). Finally, data on access to healthcare and income comes from the County Health Rankings and Roadmaps program. Specifically, we use three indicators: (1) the ratio of population to primary care physicians (2) the percentage of adults under the age of 65 without health insurance and (3) median household income.

## Empirical analysis

Our empirical analysis proceeds in two ways. We first provide a series of figures that illustrate the main results, both in terms of the relationship between density and COVID-19 deaths, the evolution of that relationship over time and the explanations behind this evolution. We then provide formal quantitative estimates for these relationships using our OLS and IV strategies. The fact that by-and-large the quantitative findings are the same regardless of the methods employed in the analysis gives us confidence on the robustness of our results to methodological decisions made in the research process.

### Graphical evidence

**Fig. 1:**
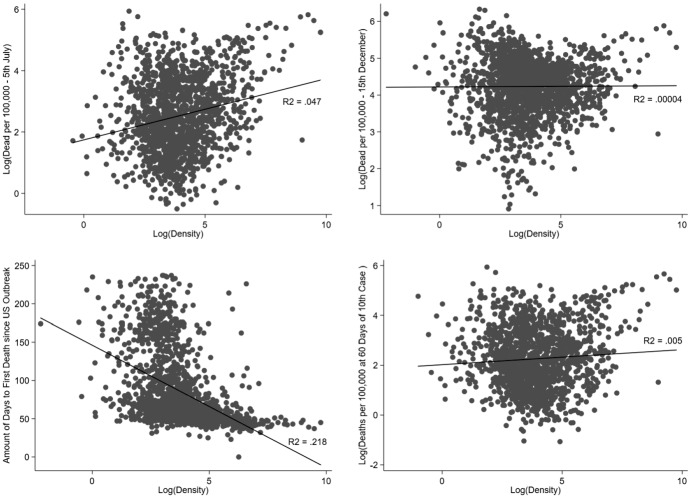
And Population Density and COVID-19 in 2020. *Notes* The horizontal axis represents the logarithm of the county’s population density. *Top left panel* vertical axis represents the logarithm of the accumulated number of fatalities per hundred thousand inhabitants by the 5th of July 2020. *Top right panel* vertical axis represents the logarithm of the accumulated number of fatalities per hundred thousand inhabitants by the 1st of December 2020. *Bottom-left panel* vertical axis represents the number of days between the 22nd of January and the first fatality in each county. *Bottom-right panel* vertical axis represents the logarithm of the number of dead 60 days after the 10th case was reported in the county. Black markers correspond to counties forming part of a CBSA. Fitted lines estimated via Ordinary Least Squares. Univariate R-squared included in all Figures alongside fitted line

The top-left panel of Fig. 1 illustrates the positive cross-sectional correlation between a county’s population density—calculated as the total population over the surface area—and the number of COVID-19- related deaths per capita by the end of the first wave on the 5th of July.^11^ This is the basic fact that had been noticed in Wheaton (2020) and Dubner (2020) as early as April 2020. Similar graphs, again displaying positive relationships using population-weighted densities and number of cases, are reported in “Appendix Fig. 7.”

Naturally, these cross-sectional patterns do not constitute conclusive evidence that urban density results in faster or more deadly COVID-19 spread. There are at least two problems that could arise in this context. First, the positive correlation in the top left panel of Fig. 1 can be the result of differences in the timing of the onset of the disease across locations. Second, certain location characteristics which are correlated with both density and COVID-19 spread and severity could induce a correlation in the absence of any actual causal link. We discuss this second issue in detail in the next section.

**Table 4:** Density and time-adjusted deaths in 2020 for different post-onset windows

	OLS	IV	
*(A) Log(Deaths per 100,000, 21 days after 10th case)*			
Log(population density)	$-$0.377***	$-$0.357***	$-$0.194**
	(0.044)	(0.116)	(0.076)
First-stage F-stat		20.1	67.2
Obs.	1159	1159	1141
*(B) Log(Deaths per 100,000, 30 days after 10th case)*			
Log(population density)	$-$0.240***	$-$0.234*	$-$0.072
	(0.046)	(0.130)	(0.074)
First-stage F-stat		20.4	72.8
Obs.	1264	1264	1242
*(C) Log(Deaths per 100,000, 45 days after 10th case)*			
Log(population density)	$-$0.126***	$-$0.162	$-$0.021
	(0.045)	(0.129)	(0.078)
First-stage F-stat		24.0	77.7
Obs.	1367	1367	1345
Instrument		Geological	Historical
State effects	Yes	Yes	Yes

Estimates of the effect of the natural logarithm of population density on time-adjusted COVID-19 deaths per 100,000 population. Different panels correspond to different choices of the time-adjustment windows in the dependent variable. Standard errors clustered at the CBSA level. ***${p}<0.01$, **${p}<0.05$, *${p}<0.1$

**Table 5:** Suggested mechanisms: social connectedness and behavioral responses

	OLS	IV	
*(A) Social connectedness*			
Log(population density)	0.619***	0.452***	0.372***
	(0.017)	(0.045)	(0.034)
IV F-stat		25.9	111.0
Obs.	1758	1758	1732
*(B)* $\Delta$ *Workplace-related activity*			
Log(population density)	$-$3.789***	$-$4.860***	$-$3.796***
	(0.156)	(0.478)	(0.301)
IV F-stat		19.5	56.8
Obs.	1355	1355	1336
*(C)* $\Delta$ *Retail-related activity*			
Log(population density)	$-$2.519***	$-$2.615**	$-$3.471***
	(0.325)	(1.022)	(0.641)
IV F-stat		19.7	50.4
Obs.	1289	1289	1270
*(D) Republican vote share 2016*			
Log(population density)	$-$0.050***	$-$0.009	$-$0.080***
	(0.003)	(0.011)	(0.008)
IV F-stat		25.9	111.0
Obs.	1759	1759	1733
Instrument		Geological	Historical
State effects	Yes	Yes	Yes

The main explanatory variable in all models is the natural logarithm of population density. In Panel A, we present the results for the social connectedness of a county based on Facebook’s Social Connectedness Index. Panels B and C report the results on behavioral adjustment of workplace and retail activities relative to the January baseline, respectively. Panel D features the results on votes for the Republican party in the 2016 presidential election. Column (1) corresponds to OLS estimates, and column (2) and (3) presents 2SLS estimates using the Geological and Historical instruments, respectively. In all models, we include controls for average maximum and minimum temperatures, average yearly precipitation, latitude, longitude, distance between the county centroid and the closest sea front and distance to the closest waterfront. The specifications in columns (2) and (3) add state effects. Standard errors in parenthesis are clustered at the CBSA level. **$\hbox {p}<0.01$, *$\hbox {p}<0.05$, *$\hbox {p}<0.1$

The top right panel of Fig. 1 illustrates the point on differences in the timing of the onset of the disease across locations by showing that the positive correlation between population density and COVID-19- related deaths observed in the *first-wave* becomes almost flat when we use data extending to the 15th of December 2020. We investigate the timing dimension further in the bottom left panel of Fig. 1 where we show the relationship between population density and the number of days between the 22nd of January and the first fatality in each county. The figure exhibits a clear negative relationship, indicating that dense locations experienced COVID-19 fatalities earlier than more sparsely populated locations.

We can adjust for the differences in the timing of the onset of the disease by computing the number of deaths after a fixed number of days from that onset. This is what is typically shown in cross-country comparisons of the early evolution of the pandemic. In our case, we can compute the number of COVID-19 deaths at a specified time after the outbreak started in a county. We define the start of the outbreak as the first day with 10 reported cases and compute the number of deaths 60 days after this date for all counties.^12^ The link between this time-adjusted variable and density is illustrated in the bottom-right panel of Fig. 1. The relationship is almost flat after time-adjusting, suggesting that density does not simply translate into a higher rate of COVID-19 fatalities.

**Fig. 2:**
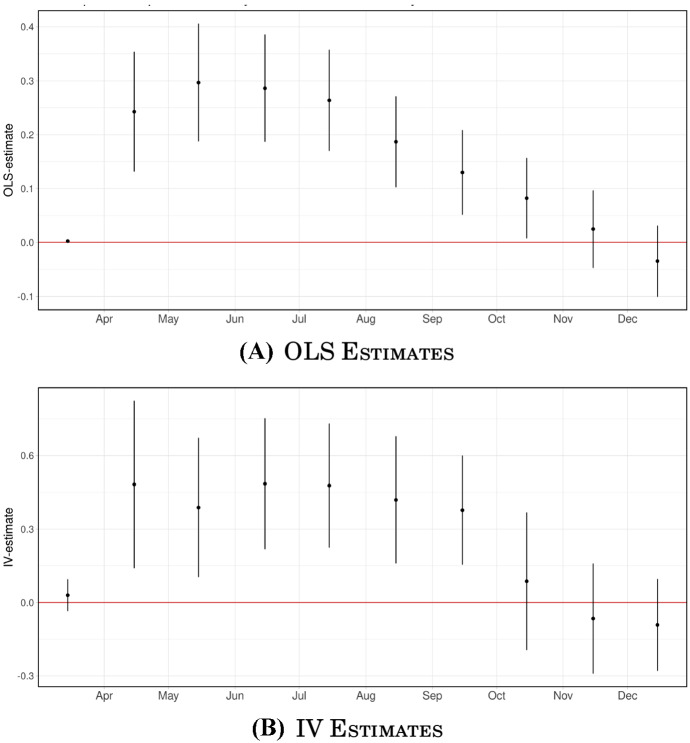
OLS Accumulated COVID-19 Deaths-Density Elasticities over Time. *Notes* Both panels depict the cross-sectional relationship between the natural logarithms of accumulated deaths and population density for every monthly period ending in the 15th, from March through December 2020. Panel A: coefficients from univariate OLS regressions. Panel B: IV estimates obtained using both the geological and historical instruments for density. For each estimate, we report the 95% confidence interval based on standard errors clustered at the CBSA level

How is it possible that initial studies reported a clear positive influence of density on the impact of COVID-19, yet we report no relationship here? The answer is illustrated in Panel A of Fig. 2, where we report how the slope of the relationship between population density and accumulated deaths evolved over 2020. These are simply the coefficients of a univariate regressions of the logarithm of total accumulated deaths—up to the period in the horizontal axis—on the logarithm of a county’s population density.^13^ Panel A of Fig. 2 shows a positive relationship between deaths and density appeared at the beginning of the US epidemic, with the positive relationship peaking by May 15th 2020. Yet in subsequent months the relationship progressively flattened, with the slopes of interest shrinking progressively until becoming statistically insignificant by November 15th. Thus, there was an apparently positive relationship at the beginning of the US epidemic, but this relationship became flat as the pandemic evolved.

**Fig. 3:**
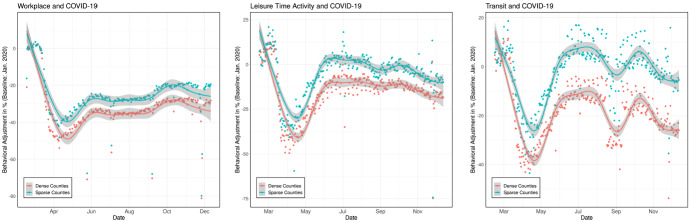
Changes in Mobility Relative to January Baseline (2020). *Notes* The figures plot the daily change and local regression curve (LOESS) over time in mobility relative to the January 2020 baseline for sparse counties and dense counties, with the split based on median weighted county density. The left panel refers to adjustment of workplace-related activity. The middle panel refers to leisure time activities including restaurants, cafes, shopping centers, theme parks, museums, libraries, and movie theaters. The right panel refers to transit including public transport hubs such as subway, bus, and train stations

**Fig. 4:**
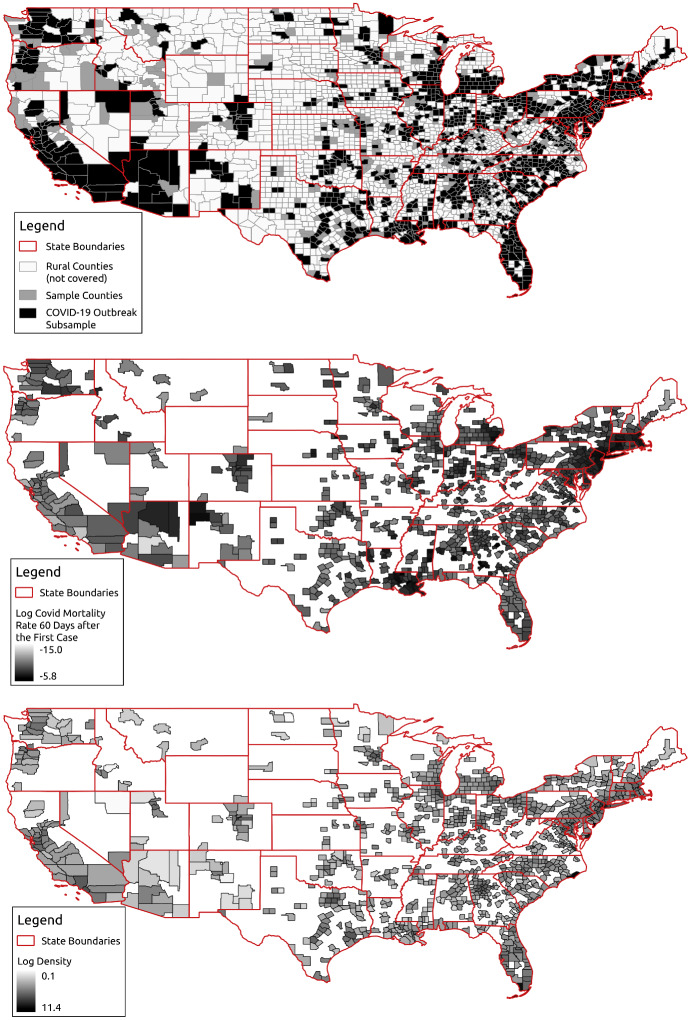
Sample counties, COVID-19 and population density

**Fig. 5:**
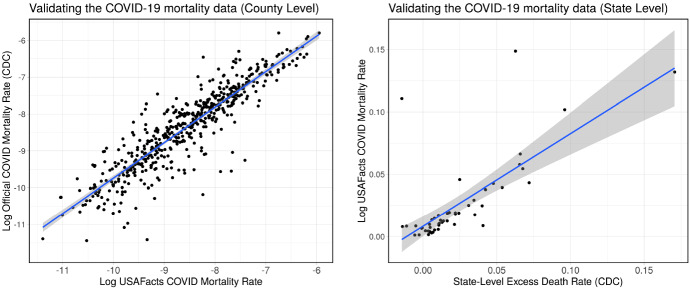
Validating Covid-19 figures. *Notes* In the left panel, the vertical axis represents the log of the officially confirmed COVID-19 mortality rate per county by the CDC and the horizontal axis the COVID-19 mortality rate by USAFacts. The right panel plots the USAFacts state-level mortality rate (vertical axis) over the excess death estimates by the CDC (horizontal axis). Blue fit lines estimated via Ordinary Least Squares including the 95% confidence interval in gray

Several factors could explain this result. We will turn to these in detail when we discuss mechanisms in Sect. 3.4, but consider as an illustration the role of changes in mobility across cities. Figure 3 shows the change in mobility relative to the January 2020 baseline for sparse and dense counties, with the split based on median county density.^14^ The left panel corresponds to changes in workplace-related mobility, the middle panel corresponds to changes in mobility for leisure activities and the right panel for transit. As expected, we observe a sharp reduction in mobility starting around mid-March. Importantly, in all cases we observe that this reduction is more acute in denser counties. Glaeser et al. (2020) show reductions in mobility had a substantial effect on the spread of COVID-19 over our sample period. Therefore, a sharper reduction in mobility in denser cities could contain the spread of the disease in these locations.

### Estimation

To obtain credible quantitative estimates of the relationship between time-adjusted COVID-19- related mortality and density, we also need to deal with potential confounders affecting both density and the prevalence and severity of the disease. Climate conditions, for example, can simultaneously influence household location decisions (see Glaeser et al. 2001) and COVID-19 spread.^15^ Local amenities such as waterfronts or low precipitation levels can themselves influence travel patterns—e.g., by increasing tourist arrivals—which could in turn affect COVID-19 rates. Insofar as some of these elements are observable, we can include them as controls in our regressions. Yet, some confounders may be unobservable due to their inherent nature or lack of accurate data. For instance, locational productive advantages can simultaneously affect local economic conditions and increase local densities.^16^ Examples range from natural factors such as fertile or irrigable lands to man-made infrastructures such as ports or highways. Insofar as COVID-19 incidence and deaths are affected by economic conditions, unobservable locational advantages can confound the effect of density on the spread and severity of the disease.

**Table 6:** Mechanisms: healthcare provision and demographics

	OLS	IV	
*(A) Log primary care physicians per capita*			
Log(population density)	0.244***	0.180***	0.148***
	(0.014)	(0.042)	(0.024)
IV F-stat		25.9	97.8
Obs.	1714	1714	1688
*(B) Share of pop. uninsured*			
Log(population density)	$-$0.003***	$-$0.005	$-$0.010***
	(0.001)	(0.003)	(0.002)
IV F-stat		25.9	111.0
Obs.	1759	1759	1733
*(C) Median houshold income*			
Log(population density)	3.975***	7.051***	2.167***
	(0.349)	(1.068)	(0.816)
IV F-stat		25.9	111.0
Obs.	1759	1759	1733
*(D) Share of pop. above 60 years*			
Log(population density)	$-$0.019***	$-$0.002	$-$0.014***
	(0.001)	(0.005)	(0.003)
IV F-stat		25.9	111.0
Obs.	1759	1759	1733
Instrument		Geological	Historical
State effects	Yes	Yes	Yes

The main explanatory variable in all models is the natural logarithm of population density. In Panel A, we present the results for primary health care supply measured as the natural logarithm of the number of primary health care physicians in each county divided by population. Panels B refers to the share of adults without health insurance. Panel C reports the results on median household income in 1000 USD. Panel D features the estimates for the share of population above 60 years of age. Column (1) corresponds to OLS estimates, and column (2) and (3) presents 2SLS estimates using the Geological and Historical instruments, respectively. In all models, we include controls for average maximum and minimum temperatures, average yearly precipitation, latitude, longitude, distance between the county centroid and the closest sea front and distance to the closest waterfront. The specifications in columns(2) and (3) add state effects. Standard errors in parenthesis are clustered at the CBSA level. **$\hbox {p}<0.01$,**$\hbox {p}<0.05$, $\hbox {p}<0.1$

To overcome the problem posed by potential unobservable confounding factors, we borrow canonical instruments for density from the agglomeration literature Combes et al. (2011) and our previous work on the relationship between density and air pollution Carozzi and Roth (2020). Specifically, we will instrument population density with either geological factors which can affect the costs of compact urban development or a long-lags in population density.

We use three geological instruments: the fraction of the urban footprint with aquifer presence, a measure of average earthquake risks and an estimate of soil drainage quality. The rationale for the aquifer instrument is that new dwellings in the periphery of urban areas need to either to pay for a costly connection with the municipal network or to directly connect with an underwater source. Given that the option of the underwater source is only available if there is an aquifer where the dwelling is located, cities with more land over aquifers can sprawl out further, contain more sparse development and lower densities. This instrument is motivated by the work in Burchfield et al. (2006) which reports that aquifers in the urban fringe are associated with urban sprawl. The rationale for our earthquake risk instrument is the expectation that the risk of an earthquake might influence building regulations, construction practices and the space between buildings, thus also affecting urban density. We also expect this instrument to satisfy the exogeneity condition, once we condition for distance to sea, average precipitation, latitude, longitude, and state fixed effects. Finally, the soil drainage quality variable is expected to affect land suitability for building at different densities. In fully urbanized land, a significant fraction of rainfall is drained through drainage networks and sewage systems (Konrad 2003). However, at lower densities, soil drainage capacity is important to avoid stagnant water and, possibly, floods. In addition, high drainage soil is not ideal for laying down heavy infrastructure, making the task of building high density development more expensive.

We use a separate instrument for density based on historical population as recorded in the 1880 US census. Settlements in this period were in place before much of the technological revolutions in transportation that have affected location patterns in the last decades and also precede current patterns of industrial location. The use of historical population instruments for density was popularized by Ciccone and Hall (1996) and has been featured recurrently in the literature on agglomeration economies since (see Combes and Gobillon 2015 for a review).

Our main estimating equation will regress measures of COVID-19 presence on the logarithm of population density:

1 $$\begin{aligned} Y_{i}=\alpha _s +\beta Ln(Pop. Density)_{i}+\gamma 'X_{i}+\varepsilon _{i} \end{aligned}$$

where *i* indexes individual counties, $\alpha _s$ is a set of state effects and $X_{i}$ is a set of controls. In all specifications, we control for average maximum and minimum temperatures, average yearly precipitation, latitude, longitude, distance between the county centroid and the closest sea front and distance to the closest waterfront. Our outcomes include different measures of COVID-19 presence. In most of our analysis, these are either variables capturing the time it took for the disease to arrive at a county or a time-adjusted measure of COVID-19 presence - the logarithm of the number of COVID-19 fatalities in the county 60 days after the 10th case was confirmed.

Before presenting our results, it is important to highlight that our estimates of parameter $\beta$ from Eq. (1) will capture the overall effect of density on the outcome of interest. This includes the effect of geographic proximity facilitating transmission but also effects operating through the impact of density on agglomeration economies, personal behavior, local population compositions, healthcare systems, etc. After reporting estimates of the overall effect of density, we will turn to investigate the specific mediating factors behind it in Sect. 3.4 below.

### Main results

We first report baseline cross-sectional correlations between population density and COVID-19 cases and deaths during the *first-wave*. In Table 2, we estimate Eq. (1) via Ordinary Least Squares (OLS) using the logarithm of the number of cases per 100,000 inhabitants and the logarithm of the number of deaths per 100,000 inhabitants as outcome variables. We find positive and statistically significant effects of population density on COVID-19 incidence, in line with the descriptive evidence reported in the top-left panel of Fig. 1. Specifically, when using the conventional measure of population density, we find elasticities of 22% and 13% for cases and deaths, respectively. This suggests that a 1% increase in population density increases cases and deaths per 100,000 people by 0.22% and 0.13%. When using our population-weighted measure of density, we also find very similar positive elasticities. The findings for COVID-19 cases are consistent with the evidence presented by Wheaton (2020) and Almagro and Orane-Hutchinson (2020). Yet this should not be taken as conclusive evidence that density has a causal effect on the spread of COVID-19. As argued above, potential differences in the timing of the onset of the disease across locations or the presence of potential unobservable confounders can induce substantial bias in these coefficients.

Estimates reported in Table 3 deal with these empirical issues by looking explicitly at differences in the onset of the COVID-19 epidemic across locations and incorporating our instrumental variable strategy. In panels A and B, we report estimates for the effect of density on the number of days to the first case and the number of days to the first death. These numbers are measured relative to the date of the first reported case in the USA, so that small numbers correspond to an earlier onset of an outbreak. In column 1, we report OLS estimates obtained after controlling for state effects and covariates. In columns 2 and 3, we show IV estimates obtained using our Geological and Historical instruments, respectively. Note that the first-stage F-stats lie at 25 or above and the instruments explain between 5% and 10% of the variance in population density, indicating that they are not weak. Our second-stage estimates confirm that denser areas have indeed experienced earlier onsets of the disease whether we use days to the first case or days to the first death. A one log-point increase in density reduces the time to the first case by between 4 and 6 days depending on the specification. The effect on the time to the first deaths is even larger. These estimates demonstrate the importance of adjusting for differences in the timing of the onsets across locations when estimating the relationship between population density and COVID-19 health outcomes.

In Panel C of Table 3, we examine our main outcome of interest; the effect of population density on time-adjusted COVID-19- related mortality. As mentioned previously, we focus on confirmed COVID-19- related deaths rather than cases as our main outcome of interest because it is considered to be a more accurate indicator of local COVID-19 prevalence. We provide a complementary analysis using reported cases in Sect. 3.5. In column 1, we find that the cross-sectional correlation observed in Table 2 becomes *negative* and statistically insignificant, suggesting that the positive link between population density and COVID-19 deaths might have been confounded by differences in the timing of the local outbreak. In columns 2 and 3, we use our instrumental variable approach to test this hypothesis more convincingly. Our second-stage results reveal a statistically insignificant relationship between population density and COVID-19- related deaths in both columns, portraying a similar picture as the OLS estimate presented in column 1. Our 2SLS results are unsurprisingly less precise, but the overall picture is clear. We find no evidence that population density is positively linked with COVID-19- related deaths.

We can use our IV strategy to reproduce the findings illustrated in Panel A of Fig. 2 showing the evolution of the cross-sectional relationship between COVID-19 deaths and population density over time. For this purpose, we estimate modified versions of Eq. (1) where the dependent variable is now the accumulated number of deaths up to the 15th day of each month in 2020 from March to December. Estimates of the different $\beta _t$ slope coefficients obtained using 2SLS are reported in Panel B of Fig. 2. In this case, we use both our geological and historical instruments as a source of exogenous variation. We observe that these results mimic those in Panel A, with an initially positive and significant relationship emerging by April 15th giving way to a progressive flatter relationship throughout 2020.

Finally, we test whether the relationship between density and time-adjusted COVID-19 deaths changes with the window used. To do this, we obtain estimates corresponding to 21, 30, and 45 day windows, all measured after the 10th case is reported in each county. The results are reported in Table 4 and show that the time-adjusted number of deaths is not positively affected by density, regardless of the window used. Interestingly, we find that at a beginning of an outbreak in a given county this relationship is in fact negative but becomes flat within two months.

On first reflection, the null (or negative) results for COVID-19 spread in this section appear surprising given that the virus spreads via human contact and denser areas can provide more opportunities for human interactions. Nevertheless, there are several mediating factors that might offset this intuitive mechanism. For example, density itself might attract younger residents who are less likely to develop significant symptoms. In addition, both behavioral and/or policy induced changes in behavior may be different in dense counties. In fact, studies on previous pandemics (e.g., the 1918 influenza pandemic) also show that population density is not necessarily linked with the spread and severity of a disease (Mills et al. 2004). In the next section, we explore potential mechanisms that can explain our reduced-form findings.

### Mechanisms

Variation in density might lead to changes in several local conditions, which can themselves affect the spread and severity of the disease. These types of changes may provide mechanisms that reinforce or offset the hypothesized positive effects that have been suggested in the literature, both in terms of timing of the local onset of the pandemic and subsequent spread. We turn to study some of these mechanisms by estimating the effect of density on other determinants of COVID-19 spread and severity. To do so, we re-estimate Eq. (1) using these hypothetical mediators as outcomes. The resulting estimates do not provide definite proof regarding the mechanisms explaining the effect of density on COVID-19 incidence and mortality, but should be interpreted as suggestive evidence in this regard.

We begin by looking at possible factors explaining the early onset of the disease in denser cities and show that density is associated with higher social connectedness with other US counties. Our proxy for this variable relies on Facebook’s Social Connectedness Index (SCI).^17^ This index is based on the relative frequency of friendship links between users of the social network, with higher index values corresponding to a larger number of friendship links. To proxy for social connectedness with other counties, we aggregate the SCI of each county with all *other* counties and normalize it by the own-county SCI. The resulting variable is large when inhabitants in a county are disproportionately connected to other counties. Coefficients resulting from estimating Eq. (1) using the logarithm of this proxy as an outcome variable are provided in Panel A of Table 5. As above, we report both OLS estimates (column 1) and 2SLS estimates using our geological and historical instruments (columns 2 and 3). We observe consistently positive elasticities of roughly 0.4-0.5 across columns, indicating denser counties are more intensely related to other counties in the USA.^18^ These results provide a plausible explanation to our findings of early onsets of COVID-19 cases and deaths in denser counties illustrated in Fig. 1 and Table 3.

Next, we study how density affects behavioral responses to the pandemic (e.g., compliance with social distancing measures). We use data from the Google COVID-19 Community Mobility Reports (CMR) to measure how mobility patterns in each county have changed relative to baseline levels measured in January 2020. In Panels B and C of Table 5, we show the relationship between county density and the change in mobility to workplaces and retail activity, respectively. We find that population density is associated with a larger decline in mobility for both indicators. Doubling density reduces workplace-related mobility and retail-related activity by approximately 2.6–3.4% and 1.7–2.4%, respectively. Given the significant variation in density across US counties, these estimates are large. Insofar as social distancing reduces the spread of the disease, these differences in behavior might explain why we find limited differences in spread by location after accounting for the timing of onset of the disease and confounding factors.

Several factors could explain this difference in behavior across dense and sparse counties. One candidate that could account for both policy responses and individual differences in behavior relates to ideological or political views. Allcott et al. (2020) show that the Republican county vote share has a positive and significant association with the number of weekly visits to points of interest during the peak of the social distancing measures in April. Anecdotal evidence also reveals substantial differences in the tone of the Democratic and Republican parties when discussing the pandemic and its consequences. If density is associated with reduced support for the Republican party, residents of denser areas may be more likely to comply with the social distancing advise. In Panel D of Table 5, we estimate this link using voting data from the 2016 presidential election as a proxy for Republican support. We find that population density has a negative association with the share of Republican voters, an observation that should come as no surprise for observers of US politics.^19^ This difference in political preferences across locations could explain, at least in part, the observed differences in the behavioral response to the pandemic illustrated in Fig. 3 and Table 5.

We can arrive at two conclusions from the results reported in Table 5. First, dense counties are more connected with other locations and this may account for earlier onset of the COVID-19 epidemic in these areas. Second, the behavioral response to the disease was larger in denser counties, with less mobility for work and leisure and reduced use of public transit in these locations.

Finally, in Table 6, we examine the effect of density on access to healthcare and demographics, as these are likely to affect COVID-19- related mortality. In Panels A and B, we examine the effect of density on access to healthcare using the ratio of population to primary care physicians and the percentage of adults under the age of 65 without health insurance as proxies. We find that density is positively associated with the former and negatively associated with the latter, suggesting that denser locations benefit from better access to healthcare. In our context, this could be an important mediating factor for two main reasons. First, access to primary healthcare might affect the presence and management of underlying health conditions which consider being risk factors for COVID-19 mortality (Zhou et al. 2020). Second, access might also affect the probability of seeking and receiving medical treatment once infected with COVID-19. Relatedly, we also examine the link between population density and income in Panel C as it is likely to affect access to healthcare and also health status more broadly. As expected, we find that the density is positively associated with median household income, offering an additional explanation for our headline results. Finally, in Panel D, we examine the effect of density on the share of the population above 60 years of age. This is of particular importance given that older age considered to be a significant risk factor (Zhou et al. 2020) and that population density is likely to affect the age structure of local areas via its impact on employment opportunities Glaeser (1999). Indeed, we find some evidence that population density is linked with a smaller share of residents above 60 years of age. In other words, dense counties are “younger” than sparse counties and this could reduce the number of deaths in these areas.

Overall, our points relating to behavioral responses, healthcare provision and demographics provide probable explanations for the surprisingly flat relationship between density and COVID-19- related mortality reported in panel C of Table 3.

### Robustness checks

In this section, we provide several tests to evaluate the robustness of our main findings. We first revisit our results for the time-adjusted COVID-19 deaths by controlling for time of onset. In Panel A of “Appendix Table 7,” we test whether the null effect of density is robust to flexibly controlling by week of onset in each state. This goes beyond simply time-adjusting the outcome variable of interest as it also incorporates differences in knowledge regarding the disease or country-wide behavioral adjustments. We find that our qualitative results remain unchanged, with coefficients being insignificantly different from 0 across specifications. In panel B, we test whether our results are affected by excluding the New York metropolitan area.^20^ In this case, we find a negative and statistically significant relationship between density and time-adjusted COVID-19 deaths in our OLS estimate but statistically insignificant effects when we use our IV methodologies. We interpret these results with caution, as we are imposing sample selection that simultaneously exclude the MSA with the largest initial outbreak and the highest density.

Much of the evidence featured in the discussion around the role of urban density in shaping the impact of COVID-19 has focused on the conventional, area-weighted definition of density (i.e., population divided by surface). In order to speak to that debate, this has been the object of our main analysis. But we can evaluate the robustness of our results to the definition of density by studying the effect of population-weighted densities. In “Appendix Table 8,” we reproduce our main results using this variable as our main independent variable of interest. Unfortunately, since our geological instruments do not provide a strong first stage for this variable, our IV analysis relies solely on our long-lag instrument. Reassuringly, we find that the overall results are qualitatively similar to those obtained in Table 3. Panels A and B show denser counties had earlier onsets of the disease compared to sparse counties. In panel C, we find a negative association between weighted density and COVID-19- related deaths when using OLS. However, our IV estimates again show a statistically insignificant elasticity. We therefore conclude that variation in density did not result in more COVID-19 incidence and deaths in the USA beyond the effect on early onset of the disease despite prior descriptive evidence. We also check the robustness of our results regarding suggested mechanisms using population-weighted density as our main regressor of interest in “Appendix Table 9.” Reassuringly, we find that the overall results are qualitatively analogous to those reported in Table 3.

Finally, we test whether density affects the time-adjusted number of reported *cases* of COVID-19. As argued above, the number of cases is more likely to be affected by variation in testing resources and by the presence of asymptomatic cases. This motivates our focus on number of deaths in much of the main analysis. In Table 10, we report estimates of the relationship between density and the number of cases per 100,000 inhabitants measured 21, 30, 45 and 60 days after the 10th reported case in the county. IV estimates for the effect of density on time-adjusted cases are similar to estimates reported in Table 4. We conclude that the data do not yield any evidence indicating a positive effect of density on the spread of the disease.

## Conclusions

Urban areas are often places of intense social interaction, crowded living and close contact. Whether Justinian’s Constantinople, fourteenth century Florence or 1918 Philadelphia - cities have historically been associated with the propagation of infectious disease. In the first three months of the global COVID-19 pandemic, large, dense urban areas around the world such as New York, Madrid and London were identified as disease hotspots. Increased awareness of the risks of present and future epidemics has understandably prompted a debate about the future of cities. Did density—the defining feature of cities—promote the spread of the disease?

Our analysis of the onset of the COVID-19 pandemic in the USA raises a series of important points regarding these questions. First, density is associated with an early arrival of COVID-19, so that urban cores and superstar cities get a head start on the spread of the disease. Second, the subsequent spread—once COVID-19 has arrived—is not faster or deadlier than in smaller towns or sparsely populated peripheries. Cities get hit first, but do not get hit harder. We argue this is one of the reasons why many of the early studies of the impact of density on the impact of COVID-19 reported positive findings. A wider look at the whole period before vaccination began yields a different overall view of this relationship.

Several mechanisms may explain these findings. Large cities are intensely inter-connected with other locations, which can explain early onset. In the case of within-city spread, different offsetting forces may be at play. Crowding may promote the spread of the disease but differences in precautionary measures, access to healthcare and demographics may contain it. As a result, our findings emphasize the importance of distinguishing between differences in spread between and within locations.

Our study contributes to the understanding of how a summary feature of urban structure—population density—shapes spread of disease and deaths. The way in which other elements or urban form, cities’ transport infrastructure or housing conditions (e.g., overcrowding) shaped the impact of the COVID-19 pandemic is not addressed here and remains an active area of research (see e.g., Kamis et al. 2021; Borsati et al. 2022 and Brotherhood et al. 2022).

## Figures and Tables

**Fig. 6:**
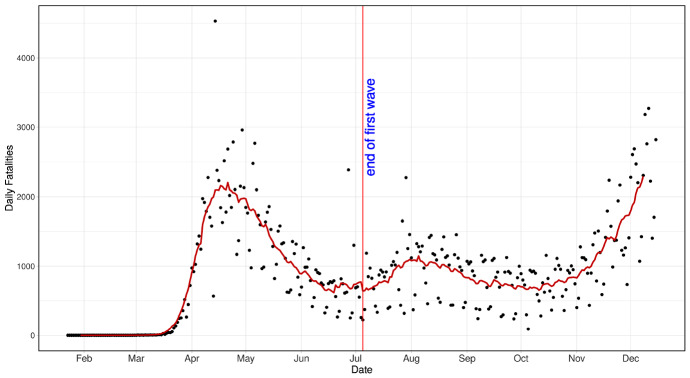
Aggregate Daily Number of COVID-19 Deaths (2020). *Notes* Daily COVID-related deaths reported in the USA between February and the 15th of December 2020. Solid line represents moving average of daily deaths

**Fig. 7:**
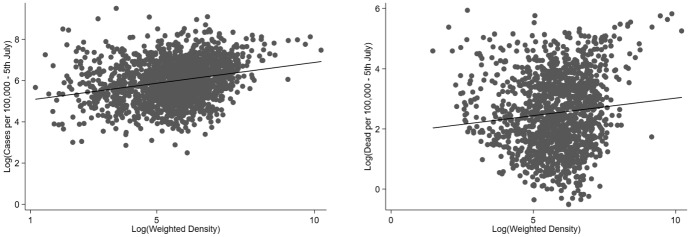
Cases and Deaths per 100,000 in First-Wave versus Weighted Density. *Notes* The horizontal axis represents the logarithm of the county’s population-weighted density. In the left panel, the vertical axis represents the logarithm of the number of cases per 100,000 inhabitants. In the right panel, the vertical axis represents the logarithm of the number of fatalities per thousand inhabitants. Black markers correspond to counties forming part of a CBSA. Black fit lines estimated via Ordinary Least Squares

## A: Additional figures and tables

See Figs. 4, 5, 6, 7 and Tables 7, 8, 9, 10.

**Table 7:** Robustness: density and deaths

	OLS	IV	
*(A) Controlling for week of onset effects*			
Log(population density)	$-$0.092**	$-$0.089	0.106
	(0.046)	(0.173)	(0.103)
First-stage F-stat		23.8	60.6
Obs.	1441	1203	1181
*(B) Excluding New York state*			
Log(population density)	$-$0.128**	$-$0.053	0.043
	(0.053)	(0.125)	(0.077)
First-stage F-stat		25.6	60.5
Obs.	1203	1441	1418
Instrument		Geological	Historical
State effects	Yes	Yes	Yes

Robustness tests corresponding to Table 3 Panel C, additionally controlling for the week of the onset (Panel A) and excluding New York State (Panel B). The main explanatory variable in all models is the natural logarithm of population density. The dependent variable is the log of the number of deaths per 100,000 inhabitants in a county 45 days after the first case. Column (1) corresponds to OLS estimates, and column (2) and (3) refer to 2SLS estimates using the Geological and Historical instruments, respectively. In all models, we include controls for average maximum and minimum temperatures, average yearly precipitation, latitude, longitude, distance between the county centroid and the closest sea front and distance to the closest waterfront. The specifications in columns (2) and (3) add state effects. Standard errors in parenthesis are clustered at the CBSA level. ***${p}<0.01$, **${p}<0.05$, *${p}<0.1$

**Table 8:** Weighted densities: onset of the disease and deaths after 60 days (2020)

	OLS	IV
*(A) Days to first case*		
Log(weight. density)	$-$5.867***	$-$9.789***
	(0.573)	(1.745)
IV F-stat		27.9
R2	0.44	0.34
Obs.	1759	1733
*(B) Days to first fatality*		
Log(weight. density)	$-$15.886***	$-$30.336***
	(1.205)	(7.102)
IV F-stat		20.8
R2	0.32	0.23
Obs.	1667	1642
*(C) Log(Deaths per 100,000, 60 days after 10th case)*		
Log(weight. density)	$-$0.078**	0.090
	(0.039)	(0.160)
First-stage F-stat		14.7
R2	0.30	0.28
Obs.	1441	1418
Instrument		Historical
State effects	Yes	Yes

The main explanatory variable in all models is the natural logarithm of weighted density. Panels A and B report the estimates for the number of days to the first case and death, respectively. Panel C reports the result for the log of the number of deaths per 100,000 inhabitants in a county, 60 days after the tenth case. Column (1) corresponds to OLS estimates, and column (2) presents 2SLS estimates using the Historical instrument. In all models, we include controls for average maximum and minimum temperatures, average yearly precipitation, latitude, longitude, distance between the county centroid and the closest sea front and distance to the closest waterfront. The specifications in columns (2) and (3) add state effects. Standard errors in parenthesis are clustered at the CBSA level. ***${p}<0.01$, **${p}<0.05$, *${p}<0.1$

**Table 9:** Robustness: suggested mechanisms and weighted densities

	OLS	IV
*(A) Social connectedness*		
Log(weight. density)	0.532***	0.724***
	(0.021)	(0.091)
IV F-stat		27.7
Obs.	1758	1732
*(B)* $\Delta$ *Workplace-related activity*		
Log(weight. density)	$-$2.968***	$-$7.501***
	(0.200)	(1.248)
IV F-stat		15.6
Obs.	1355	1336
*(C)* $\Delta$ **Retail-related activity**		
Log(weight. density)	$-$1.893***	$-$7.293***
	(0.413)	(1.765)
IV F-stat		13.0
Obs.	1289	1270
*(D)* *Republican vote share 2016*		
Log(weight. density)	$-$0.048***	$-$0.156***
	(0.004)	(0.023)
IV F-stat		27.9
Obs.	1759	1733
Instrument		Historical
State effects	Yes	Yes

Corresponds to Table 5, using the log of weighted density as the main explanatory variable

**Table 10:** Robustness: cases

	OLS	IV	
*(A) Log(cases per 100,000, 21 days after 10th case)*			
Log(population density)	$-$0.249***	$-$0.334***	$-$0.148**
	(0.033)	(0.110)	(0.059)
IV F-stat		25.9	111.0
Obs.	1759	1759	1733
*(B) Log(cases per 100,000, 30 days after 10th case)*			
Log(population density)	$-$0.187***	$-$0.269**	$-$0.076
	(0.035)	(0.117)	(0.063)
IV F-stat		25.4	109.8
Obs.	1758	1758	1732
*(C) Log(cases per 100,000, 45 days after 10th case)*			
Log(population density)	$-$0.133***	$-$0.200*	$-$0.047
	(0.035)	(0.116)	(0.066)
IV F-stat		25.3	109.5
Obs.	1757	1757	1731
*(D) Log(cases per 100,000, 60 days after 10th case)*			
Log(population density)	$-$0.107***	$-$0.145	$-$0.023
	(0.035)	(0.111)	(0.064)
IV F-stat		25.6	108.6
Obs.	1754	1754	1728
Instrument		Geological	Historical
State effects	Yes	Yes	Yes

The dependent variables are the log of the number of cases 60 days per 100,000 inhabitants after the 10th confirmed case. Column (1) corresponds to OLS estimates, and column (2) and (3) refer to 2SLS estimates using the Geological and Historical instruments, respectively. In all models, we include controls for average maximum and minimum temperatures, average yearly precipitation, latitude, longitude, and the distance between the county centroid and the closest sea front. The specifications in columns (2) and (3) add state effects. Standard errors in parenthesis are clustered at the CBSA level. ***${p}<0.01$, **${p}<0.05$, *${p}<0.1$

## B: Data sources

- *USAfacts.org COVID-19 Data*
  USAFacts is a non-profit civic initiative that provides data on the US population and government and works in partnership with the Penn Wharton Budget Model and the Stanford Institute for Economic Policy Research (SIEPR). The data can be retrieved at: https://usafacts.org/visualizations/coronavirus-covid-19-spread-map/. [Last visited: December 18th 2020]
- *CDC Official COVID-19 Mortality Rate* This database comprises confirmed or presumed COVID-19 fatalities and is limited to counties with at least 10 COVID-19 deaths. It should be noted, the dataset is incomplete because of the time lag between the death and the official certificate submitted to the National Center for Health Statistics (NCHS). For this reason, these data correspond to 514 counties only. The latest figures can be downloaded at: https://data.cdc.gov/NCHS/Provisional-COVID-19-Death-Counts-in-the-United-St/kn79-hsxy. [Last visited: December 18th 2020]
- *CDC Excess Mortality* Excess mortality corresponds to the deviation of total deaths to average expected deaths based on the experience in past years for each state. The latest estimates can be downloaded at: https://www.cdc.gov/nchs/nvss/vsrr/covid19/excess_deaths.htm. [Last visited: December 18th 2020]
- *US Census* contains information about demographics on the country level and can be accessed via: https://www.census.gov/data/tables/time-series/demo/popest/2010s-counties-detail.html. [Last visited: May 14th 2020]
- *‘COVID-19 Community Mobility Reports’ by Google*
  This report contains information about the behavioral activity change and social distancing in response to the COVID outbreak by county and day. For more detail on this database please visit https://www.google.com/covid19/mobility/data_documentation.html?hl=en. [Last visited: December 18th 2020]
- *Social Connectedness Data* Obtained after presenting a brief email application for the data based on this paper’s outline to Mike Bailey and others at Facebook. April 6 2020 Release Version.
- *Healthcare and Income Data from The County Health Rankings and Roadmaps program* contains information on healthcare access and various social and economics indicators at the country level and can be accessed via: https://www.countyhealthrankings.org. [Last visited: July 3rd 2020]

## Footnotes

1 See Duranton and Puga (2020), Voigtländer and Voth (2013) for treatments of this relationship in economics.

2 The attribution of detrimental effects of density for the evolution of the epidemic was not specific to the USA. On 9 of December 2020, Michael Gove (Chancellor of the Duchy of Lancaster and Minister for the UK Cabinet Office) said on ITV’s Good Morning Britain that population density was one of the reasons why the UK had more COVID-19- related deaths in comparison to Germany.

3 See for example Angel et al. (2020), Whittle and Diaz-Artiles (2020), Zhang and Schwartz (2020), Wheaton (2020) and Almagro and Orane-Hutchinson (2020). For a review of the empirical literature on the topic—covering papers in urban planning, economics and medical sciences—see Teller (2021).

4 For example, there is evidence that a higher percentage of overcrowded households and poor housing conditions in US counties have both lead to higher mortality from COVID-19 (Ahmad et al. 2020; Krieger et al. 2020; Kamis et al. 2021).

5 The literature on the relationship between the 1918 Influenza pandemic (the Spanish Flu) and population density is naturally more developed and can shed light on the link between pandemics and density more broadly. Interestingly, while it may seem intuitive that the influenza pandemic was positively associated with population density as the virus spread via human contact, a review of the literature produce mixed results. For example, Garrett (2007) finds a positive relationship between mortality rates and population density in the USA. In contrast, Mills et al. (2004) find no statistical association between population density and the initial reproductive number (R) using data on 45 US cities. Chowell et al. (2008) also find no association between transmissibility, death rates and indicators of population density in England and Wales. Ferguson et al. (2006) studies the development of the 1918 pandemic and finds evidence for an early onset in dense urban cores before a more smooth development of the disease across space.

6 Urban counties are those that are classified as either ‘metropolitan’ or ‘micropolitan’ core-based statistical areas in the 2010 census.

7 These are obtained from county-level reports by local health authorities across the USA. See “Appendix B” for further details.

8 Recent work led by Diego Puga looks at the relationship between density and COVID-19 incidence in Spain using prevalence data obtained from randomized serological tests. Cross-sectional correlations using this information point to a flat (or weakly negative) relationship between the disease’s spread and density.

9 In contrast, the correlation between county-level COVID-19 fatalities and USAFacts is −0.001 and insignificant indicating that COVID-19 mortality is not simply an amplification of fatalities occurring under normal circumstances but rather follows distinct patterns that are consistently capture by our database.

10 Note that, while the assumption of uniform distribution is clearly a simplification which could lead to measurement error, this should not have a substantial impact on our main estimates. This is because measurement error in the instruments could affect the relevance of the instruments but should not generate bias in the coefficients of interest unless the measurement error itself is correlated with COVID-19 incidence.

11 We define the first wave as the period between the onset of the disease in the USA in February 2020 and the minimal daily death rate before the second rise in COVID-19 fatalities. See “Appendix Fig. 6.”

12 The choice of 10 cases as marking the start of an outbreak from which we take the 60-day window is taken so as to ensure that there is some degree of within-county transmission at the time the window starts. We study how results change using different post-onset time windows in Sect. 3.2.

13 Specifically, we estimate $Ln(\text {Acc. Deaths}^t_{i}+1)=\alpha _0+\alpha _t Ln(Pop. Dens_{i})+\varepsilon _i$, where *i* is an index for counties and *t* indicates the end period, so that $\text {Acc. Deaths}^t_{i}$ corresponds to accumulated deaths in county *i* from the start of the pandemic up to date *t* (e.g., the 15th of April).

14 The data are based on COVID-19 Community Mobility Reports released by Google and is based on data from portable device users in US counties.

15 A number of recent papers document a negative effect of temperature on COVID-19 incidence, at least in temperate weathers. See for example Prata et al. (2020), Tobías and Molina (2020).

16 Locational advantages increase local densities because higher land prices in these areas trigger a substitution of land for capital in the production of structures (i.e. an increase in building heights).

17 Kuchler et al. (2020) study how interpersonal networks provided a channel for the spread of the disease based on the SCI.

18 Dense counties are also candidates to have higher connectedness with locations outside of the USA.

19 This relationship remains highly robust upon controlling for the share of black population as well as the population above 60 years of age. In fact, when adding these additional controls, the relationship remains between -0.04 and -0.05 and significant at the 99% confidence level for all three estimation approaches.

20 We use the census 2010 definition corresponding to the New York-Northern New Jersey-Long Island CBSA.
